# Impact of compression stockings on leg swelling after arthroscopy – a prospective randomised pilot study

**DOI:** 10.1186/s12891-019-2540-1

**Published:** 2019-04-09

**Authors:** Tina S. Tischer, Sebastian Oye, Robert Lenz, Peter Kreuz, Wolfram Mittelmeier, Rainer Bader, Thomas Tischer

**Affiliations:** 1 Department of Cardiology, University Medicine Rostock, Rostock, Germany; 2 Department of Urology, Asklepios Klinik Barmbek, Hamburg, Germany; 3 Department of Orthopedics, University Medicine Rostock, Doberaner Str. 142, 18057 Rostock, Germany; 4 Department of Orthopedic Surgery, Asklepios Clinic Bad Tölz, Bad Tölz, Germany

**Keywords:** Post-operative swelling, Knee arthroscopy, Complications, Compression therapy

## Abstract

**Background:**

Post-operative limb swelling may negatively affect the outcome of arthroscopic surgery and prolong rehabilitation. The aim of this pilot study was to evaluate the effect of compression stockings versus no compression on post-operative swelling and pain in the early post-operative phase.

**Methods:**

A single-centre, randomised controlled trial was performed. Patients who underwent minor knee arthroscopy were randomised to wear class II compression stockings (23-32 mmHg) (CS) or no compression stockings (NCS) immediately post-operatively for ten days. All patients received low molecular weight heparin (LMWH) at prophylactic dosage. The primary outcome variable was post-operative swelling of the limb, quantified by using an optical 3D measurement system (Bodytronic© 600). Pain was rated on a visual analogue scale (VAS). From a total of 76 patients assessed, 19 patients were eligible for final analysis. The trial followed the CONSORT criteria, was registered at clinicaltrial.gov and approved by the local ethics committee.

**Results:**

The circumference at the middle thigh (cF) was significantly different between groups at day 10 (*p* = 0.032; circumference − 1.35 ± 2.15% (CS) and + 0.79 ± 3.71% (NCS)). Significant differences were also noted around the knee (cD) at day 10 (*p* = 0.026) and a significant trend at cD and at the mid lower leg (cB1) at day 4. The volume of the thigh was also different with marked difference between days 1 and 4 between the two groups (*p* = 0.021; volume + 0.54 ± 2.03% (CS) and + 4.17 ± 4.67 (NCS)). Pain was lower in compression group (not statistically significant).

**Conclusions:**

Post-operative limb swelling can be reduced significantly by wearing compression stockings in the early post-operative phase when compared to not wearing stockings. This may improve the rehabilitation process after arthroscopic surgery. The optimal duration of compression therapy seems to be between three and ten days.

**Trial registration:**

clinicaltrials.gov (NCT02096562, date of registration 11.11.2013).

## Background

Arthroscopy of the knee is a common orthopaedic surgical procedure in patients with traumatic and degenerative conditions and is widely performed in industrialised countries [1]. The advantages of this minimally invasive surgery are less pain and earlier restoration of function as well as less swelling compared to open surgery. Major complications are rare, but haemarthrosis, swelling and lymphoedema may affect the patient in the early post-operative period and lead to prolonged rehabilitation [2]. Cold therapy, compression therapy, immobilisation, elevation of the affected limb, and other measures can be performed to reduce post-operative swelling [3–6].

To date, few prospective clinical trials deal with the effects of compression therapy on post-operative leg swelling, particularly after knee arthroscopy, despite its high prevalence [7]. Furthermore, the exact measurement of circumference and volume of the lower limb is difficult [8]: water plethysmography can measure the lower leg volume exactly, but no correlation with the location of the swelling can be derived [9]. Magnetic resonance imaging (MRI) can reliably measure circumference and volume, but its application is time consuming and expensive, it is also not routinely applicable to all patients (e.g. with cardiac pacemakers) [10]. Computer tomography (CT) provides exact data but is associated with harmful radiation. Advances in 3D imaging technology, however, enable reliable measurements of the volume of the lower extremity to be made in less than one minute using optical methods, allowing valid and reliable investigation of post-operative limb swelling (Tischer T, Oye S, Feldhege F, Jacksteit R, Mittelmeier W, Rainer B, et al: Measuring lower limb circumference and volume – introduction of a novel optical 3D volumetric measurement system, submitted; [11, 12]).

Therefore, we investigated the effect of compression stockings (CS) on post-operative limb swelling, oedema, and pain after outpatient knee arthroscopy in a prospective randomised trial and compared the study group to patients treated without compression stockings (NCS).

## Methods

### Study design and population

The clinical trial was approved by the Ethical Review Committee of the University of Rostock (A 2013–0083) and registered using clinicaltrials.gov (registration number NCT02096562, date 11.11.2013).

**Fig. 1:**
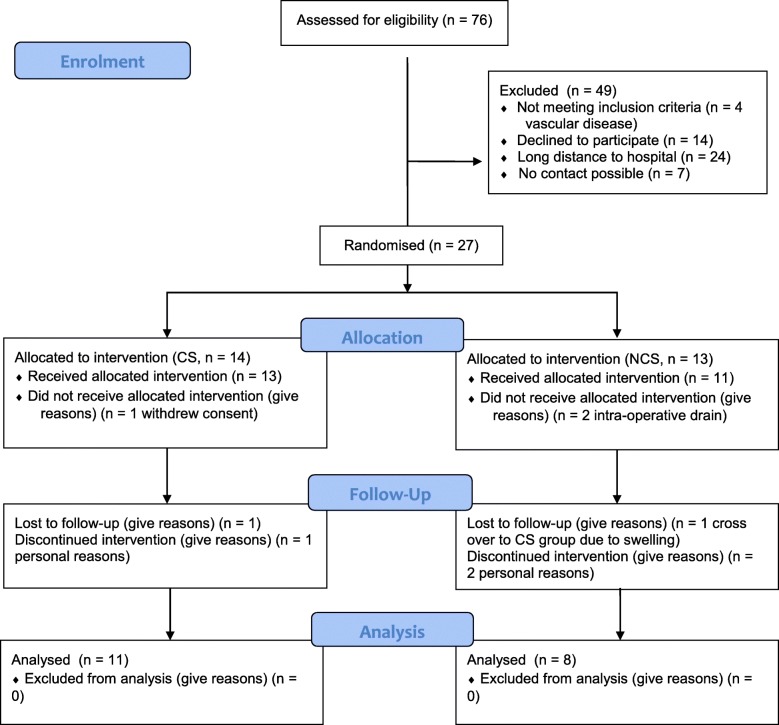
CONSORT flow chart

Eligible participants were patients undergoing minor outpatient knee arthroscopy (meniscectomy, cartilage surgery, limited partial synovectomy, no intra-articular drain after surgery) in an academic hospital and older than 18 years. Exclusion criteria were body mass index (BMI) < 18.5 or > 40 kg/m^2^, internal illness involving the cardio-vascular or endocrine system, as well as persons with liver and/or kidney malfunctions and those with phlebo-thrombotic and arterial obstructive diseases of the legs, and patients taking oedema-influencing drugs. Non-steroidal anti-inflammatory drugs were allowed. Written informed consent was obtained from all participants prior to participation. Eligible patients were randomly assigned to one of two groups before surgery using blocked randomisation by a computer-generated table of random numbers, a block size of four, and an allocation ratio of 1:1. The orthopaedic surgeon was blinded concerning the patient group. Participants were sequentially allocated to wear 3D-fitted compression stockings class II (23-32 mmHg, VenoTrain soft®, Bauerfeind AG, Zeulenroda, Germany) (CS, *n* = 14) or to the control group without compression therapy (NCS, *n* = 13) after knee arthroscopy. Finally, 11 of 14 patients in group CS and 8 of 13 people in group NCS completed the trial (Fig. 1). Following arthroscopic knee surgery, all patients received low molecular weight heparin (LMWH) at prophylactic dosage and compressive wrappings for 24 h. After that, patients in the CS group were requested to wear the compression stockings for at least eight hours a day whereas patients of the NCS group were not allowed to continue compression therapy. Additionally, patients were asked to complete a basic activity record (lying, sitting, or standing in h/day). Blinded follow up examinations were performed at 1, 4, and 10 days after surgery.

### Volume and circumference measurement

**Fig. 2:**
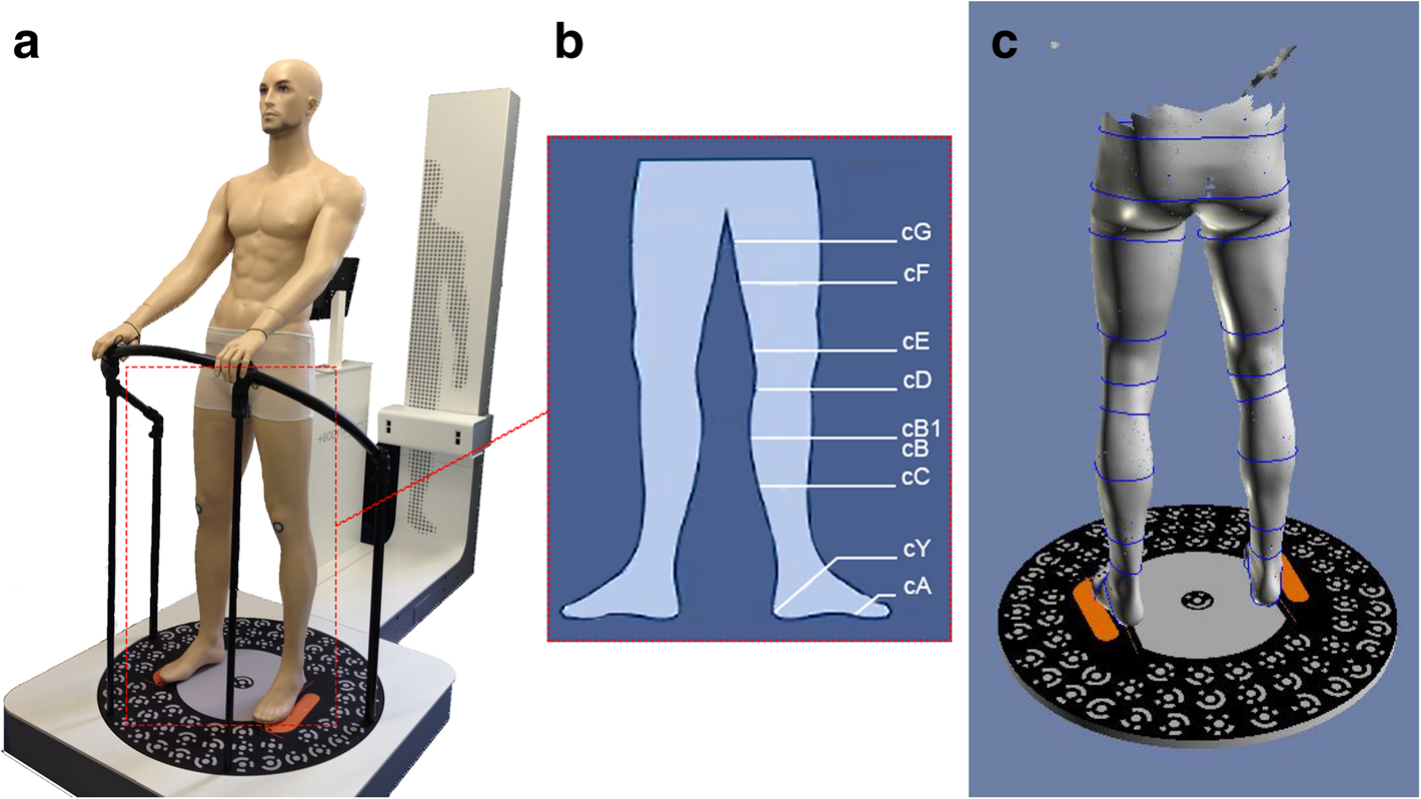
**a**) measuring device (Bodytronic© 600, Bauerfeind AG, Zeulenroda, Germany) **b**) circumference measurements c) generated 3D Model used for measurements

Primary outcome parameters were circumference and volume of the lower extremity at different time points (Fig. 2). At each time point volume and circumference measurements were performed using optoelectronic measurements (Bodytronic© 600, Bauerfeind AG, Zeulenroda, Germany) (Tischer T, Oye S, Feldhege F, Jacksteit R, Mittelmeier W, Rainer B, et al: Measuring lower limb circumference and volume – introduction of a novel optical 3D volumetric measurement system, submitted). In brief, patients were requested to remove the stockings, wait for 30 min to allow the extremity to normalise blood flow and swelling, and then positioned on the rotating measurement platform of the optoelectronic device (Fig. 2). During scanning a point cloud based on the body surface is generated and transformed into an anatomical, 3-dimensional, digital model. The circumference of various locations of the lower extremity (according to RAL, German Institute for Quality Assurance and Qualifications [13]) and the volume of the upper and lower leg were also measured. Optical scanning took less than one minute (Tischer T, Oye S, Feldhege F, Jacksteit R, Mittelmeier W, Rainer B, et al: Measuring lower limb circumference and volume – introduction of a novel optical 3D volumetric measurement system, submitted).

### Secondary outcome parameters

Secondary outcome parameters were pain in the operated leg recorded on a visual analogue scale (VAS) and range of motion (ROM) of the knee joint. To evaluate post-operative pain, participants in both groups were requested to quantify their discomfort by using a VAS. This is a reliable, valid, and frequently-used tool for pain outcome measurement [14]. The scale is composed of a straight line with two labels, that is, “no pain” and “worst possible pain”, located at either end of the line. On days 1, 4, and 10 all patients were instructed to mark on the line the point value reflecting their level of pain.

### Statistical analysis

Statistical analysis was performed using SPSS V23 software (IBM, Armonk, USA). Sample size calculation was not possible, since no valid quantitative data for swelling after knee arthroscopy were available. Therefore, we performed a pilot study. The results of our present study will help to perform adequately powered studies on this topic in the future. Besides descriptive statistics, evaluation of significant changes between the groups was performed using the non-parametric Friedman-Test (2-sided, α = 0.05) and post-hoc analysis with the Wilcoxon-Test to analyse changes between the days. Percental changes between both groups in each corresponding time frame were evaluated with the non-parametric Mann-Whitney-U-Test (2-sided, α = 0.05) and the asymptotic significance (p) was calculated as group difference. Results where *p* ≤ 0.05 were described as significant, and *p* < 0.1 was characterised as a significant trend.

## Results

Of the 76 eligible patients, 27 patients were recruited (35.5%) (Fig. 1). The major reason for not participating in the study was long distance to the hospital (*n* = 24, 32%). Fourteen patients were randomised to undergo compression therapy (CS) after knee arthroscopy and 13 patients were randomised to be treated without compression. During the trial, three patients (21.4%) of the CS group had to be excluded: one patient because of non-compliance, another was lost to follow up and the third withdrew consent (Fig. 1). Eight patients of the NCS group completed the trial as two withdrew consent, one participant changed to the CS group due to severe swelling, and two patients received drainage during knee arthroscopy and were excluded from the study. Ultimately, the NCS group consisted of 8 patients (mean age 52.8 ± 10.9 years, BMI 28.9 ± 3.4 kg/m^2^ 3.4, 6 men and 2 women) and the CS group of 11 patients (mean aged 55.0 ± 8.8 years, BMI 27.0 ± 5.0 kg/m^2^, 5 men and 6 women) (Table 1). Arthroscopic surgery was mainly performed on the right side in both groups (Table 1). No difference in physical activity was noted between the groups.

**Table 1:** Patient demographics

Parameter	Total (*n* = 19)	Group CS (*n* = 11)	Group NCS (*n* = 8)
Age (y), SD	53.7 (9.6)	55.0 (8.8)	52.8 (10.9)
Height (m), SD	1.73 (0.1)	1.77 (0.1)	1.71 (0.1)
Weight (kg), SD	84.8 (15.6)	84.5 (18.0)	85.1 (15.3)
BMI (kg/m2), SD	28.1 (4.0)	27.0 (5.0)	28.9 (3.4)
Male	11	5	6
Surgery right knee	15	9	6

*CS* compression stockings, *NCS* no compression stockings

**Fig. 3:**
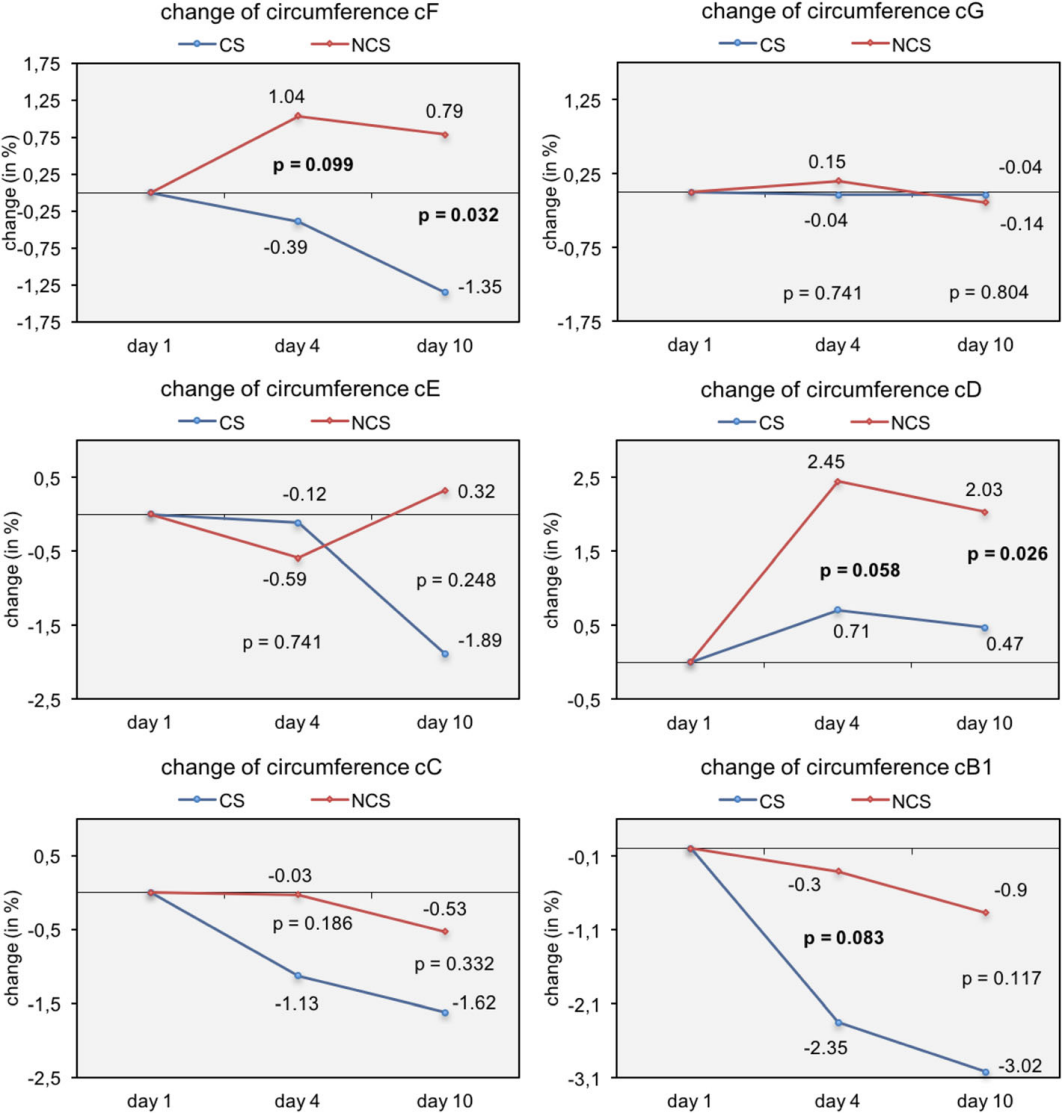
Average change (%) in circumference at different measuring points after surgery in group with compression stockings (CS) and no compression stockings (NCS)

The patients in the CS group wore the compression stockings for mean 21.73 ± 5.19 h on the first day, for 22.0 ± 4.26 h on the second day, and for 9.36 ± 0.68 h for the other eight days. The circumference at the middle of the thigh (cF) was statistically significantly different between the groups at day 10 (*p* = 0.032) (Fig. 3). In the CS group limb volume was reduced by 1.35%, whereas it increased by 0.79% in the NCS group (Table 2). Statistically significant differences in circumference of the operated leg were also noted around the knee (cD) at day 10 along with a significant trend at day 4 for cD and cB1 (Fig. 3). The volume of the thigh was also significantly different between days one and four between the two groups (*p* = 0.021; + 0.54 ± 2.03% in group CS and + 4.17 ± 4.67 in group NCS) (Table 2).

**Table 2:** Volume changes

Parameter	Group CS (*n* = 11) MW (SD) [%]	Group NCS (*n* = 8) MW (SD) [%]	p
Change between day 1 and 10 (post surgery)			
UL	- 0.96 (3.81)	2.73 (5.34)	0.099
LL	- 1.70 (3.72)	0.58 (5.30)	0.322
Change between day 1 and 4 (post surgery)			
UL	0.54 (2.03)	4.17 (4.67)	0.021
LL	- 1.46 (2.79)	0.90 (3.07)	0.117
Change between day 4 and 10 (post surgery)			
UL	- 1.51 (3.37)	- 1.44 (3.53)	0.869
LL	- 0.24 (2.82)	- 0.32 (3.82)	0.869

Changes in volume (in %) of the lower (LL) and upper lower (UL) limb between different days in groups CS (compression stockings) and NCS (no compression stockings)

Pain was lower in the compression group and significantly reduced from day 1 to 10 in this group (*p* = 0.001, VAS 1.73 ± 1.46 on day 1 to 0.47 ± 0.47 on day 10) (Table 3). Pain also decreased in NCS group up to day ten (VAS 2.51 ± 2.18 on day 1 to 1.34 ± 1.25 on day 10). However, comparing the groups, no significant difference in post-operative pain was found (*p* > 0.1) (Table 3). ROM improvement of the knee joint was not different between the groups in the early post-operative phase (at day 10 CS patients achieved 95,61% of preoperative active flexion and NCS group achieved 88,1% of preoperative active flexion).

**Table 3:** Pain development

Parameter	Group CS (*n* = 10) Av (SD)	Group NCS (n = 8) Av (SD)	p (r)
Pain on VAS			
p.s. 1	1.73 (1.45)	2.51 (2.18)	0.639 (0.119)
p.s. 4	1.00 (0.82)	1.55 (1.42)	0.464 (0.184)
p.s. 10	0.47 (0.47)	1.34 (1.25)	0.107 (0.382)

*AV* Average, *CS* compression stockings, *NCS* no compression stockings, *p.s*. post surgery. (One patient in group CS was excluded because of non-compliance with pain medication)

## Discussion

This pilot study is, to the best of the authors´ knowledge, the first randomised clinical trial addressing the therapeutic effect of compression stockings (23-32 mmHg) on leg swelling following knee arthroscopy compared with no compression therapy. The observation period incorporated the first ten post-operative days. Analysis of our data showed that wearing compression stockings in the early post-operative phase reduced post-operative leg swelling significantly. Moreover, reduced pain was also noted between the groups, although this was not statistically significant.

Our results are comparable with other studies investigating post-operative swelling after total knee arthroplasty [5, 15, 16]. Conversely, Munk et al. found no significant difference in post-operative leg swelling after total knee arthroplasty, whether using compression stockings or not [5]. Munk et al.’s results may be influenced by their use of a tape measure, as measurement errors with this method were reported by Jakobsen et al. [17]. Further, the non-significant results reported by Munk et al. [5] may have been influenced by only performing measurements at knee level and below: we detected most swelling in the thigh, in line with other comparable trials [15, 16, 18]. In contrast to these trials, we revealed reduced swelling when wearing CS, using newer measurement techniques. Pichonnaz et al. also noted massive swelling after total knee arthroplasty within the first two days using bioimpedance spectroscopy [15]. In our study cohort swelling increased up to four days after surgery without compression. Gao et al. and Stocker et al. also reported maximum thigh swelling between the second and fifth day [16, 18]. Furthermore, Brock et al. performed a prospective randomised study of the effect of inelastic compression bandages on swelling after total knee arthroplasty compared with standard wool and crepe bandages [3]. No differences between the two bandages were noted, however, in this clinical trial swelling was again only measured by tape. As reported by Holm et al. [19] increased knee circumference after total knee arthroplasty correlated significantly with decrease in knee-extension strength and slower rehabilitation. Additional studies outside of orthopaedic surgery have shown the positive effect of wearing compression stockings, e.g. after vein surgery, with reduced swelling and pain [20].

Moreover, mechanical methods such as graduated compression stockings have traditionally been used for prophylaxis of deep vein thrombosis (DVT) after surgery because they have been shown to decrease the risk of DVT [21, 22]. However, this has been regarded as controversial in guidelines for venous thromboembolism risk assessment and prevention still differs between countries and societies and several trials show mixed results [23].

Mauck et al. did not recommend thromboprophylaxis after minor knee arthroscopy because the incidence of symptomatic VTE (venous thromboembolism) after knee arthroscopy was quite low (0.4% at six weeks) [24]. Maletis et al. also showed, in a broad retrospective study with a patient cohort of 20,770, low incidence of symptomatic VTE after elective knee arthroscopy [25]. Furthermore, a recently published meta-analysis [1] presented low incidence of DVT, but statistical analysis even showed no effectiveness of anticoagulants for thromboprophylaxis in preventing DVT in patients undergoing minor knee arthroscopy. In contrast, Sun et al. described a relatively high incidence of VTE diagnosed with venography after arthroscopic knee surgery of 14.9%, (11.2% asymptomatic) with advanced age and complex arthroscopic surgery being strongly associated with VTE [26]. But even in patients who had already suffered a DVT, the effect of compression stockings versus no compression in addition to LMWH therapy did not lead to significant additional benefits after three weeks of therapy. When worn for one week, CS supported thrombus regression significantly faster [27]. Overall, the reported incidence of DVT without prophylaxis following arthroscopic knee surgery varies from 0.2 to 18%, but agreement on how to perform prophylaxis following knee arthroscopy is still under debate [28]. Compression stockings might be advantageous for VTE prophylaxis in selected surgical patients [29], but this was not the focus of our present study.

Most current studies evaluating post-operative swelling are limited by the measurement technique used [3, 5]. CT and MRI provide accurate measures of limb volume and a further differentiation of bone, adipose tissue, and muscle tissue [10, 11, 30]. However, these methods are expensive, time-consuming, and involve exposure to radiation in the case of CT, which makes these techniques difficult to use for research questions. Water displacement volumetry, still the gold standard for determining limb volume, may produce major errors, as volume changes between repeated measurements of > 30 ml have been found (Tischer T, Oye S, Feldhege F, Jacksteit R, Mittelmeier W, Rainer B, et al: Measuring lower limb circumference and volume – introduction of a novel optical 3D volumetric measurement system, submitted). Rabe et al. [9] advised that short-term repeated measurements of volume should be between 10 and < 20 ml because a volume change of > 30 ml is considered to be clinically relevant. Our results showed even lower volume changes of 0.5 to 5.0 ml between repeated measurements when subjects were repositioned, indicating high reliability (Tischer T, Oye S, Feldhege F, Jacksteit R, Mittelmeier W, Rainer B, et al: Measuring lower limb circumference and volume – introduction of a novel optical 3D volumetric measurement system, submitted).

## Limitations of the study

In order to simulate the clinical situation in which all knee arthroscopies received compression for 24 h also the non-compression group received compression for 24 h to avoid massive swelling. Despite this, we could prove an additional effect of compression stockings on swelling after 24 h of compression after arthroscopic knee surgery. However, clinical differences were small.

The study began 24 h after surgery and standard compression in both groups, which had the additional advantage that fluid (water) extravasation after arthroscopy was likely resorbed.

Although the follow-up period was only ten days, since swelling was the main outcome measurement this amount of time was sufficient to investigate early post-operative swelling in the two groups. We had a consistent result while compression stockings were worn, but we had neither information about the activity levels of the patients nor time spent with legs raised, which also influences the amount of swelling. Also thrombosis prophylaxis with LMWH may have increased swelling in both groups.

Furthermore, a limited number of participants (from 76 screened only 19 patients remained for final evaluation) was finally analysed due to the strict inclusion criteria and due to long distance drive of patients to the hospital (*n* = 24, 32%). However statistical analysis showed significant effects.

## Conclusion

Post-operative swelling of the limb can be reduced significantly by wearing 3D-fitted compression stockings class II in the early post-operative phase. The optimal duration of compression therapy seems to be between three and ten days. Additional positive effects on prophylaxis of thrombosis may be present.

## Abbreviations

CS: compression stockings
DVT: deep vein thrombosis
LMWH: low molecular weight heparin
NCS: no compression stockings
VTE: venous thrombembolism

## References

[1] Zheng G, Tang Q, Shang P, Pan XY, Liu HX (2018). No effectiveness of anticoagulants for thromboprophylaxis after non-major knee arthroscopy: a systemic review and meta-analysis of randomized controlled trials. J Thromb Thrombolysis.

[2] Bohensky MA, deSteiger R, Kondogiannis C, Sundararajan V, Andrianopoulos N, Bucknill A (2013). Adverse outcomes associated with elective knee arthroscopy: a population-based cohort study. Arthroscopy..

[3] Brock TM, Sprowson AP, Muller S, Reed MR (2017). STICKS study - short-sTretch inelastic compression bandage in knee swelling following total knee arthroplasty - a feasibility study. Trials..

[4] Song M, Sun X, Tian X, Zhang X, Shi T, Sun R (2016). Compressive cryotherapy versus cryotherapy alone in patients undergoing knee surgery: a meta-analysis. Springerplus..

[5] Munk S, Jensen NJ, Andersen I, Kehlet H, Hansen TB (2013). Effect of compression therapy on knee swelling and pain after total knee arthroplasty. Knee Surg Sports Traumatol Arthrosc.

[6] Kayamori S, Tsukada S, Sato M, Komata K, Isida Y, Wakui M (2016). Impact of postoperative compression dressing using polyethylene foam pad on the multimodal protocol for swelling control following total knee arthroplasty: a randomized controlled trial. Arthroplast Today.

[7] Gatewood CT, Tran AA, Dragoo JL (2017). The efficacy of post-operative devices following knee arthroscopic surgery: a systematic review. Knee Surg Sports Traumatol Arthroscopy.

[8] Brodovicz KG, McNaughton K, Uemura N, Meininger G, Girman CJ, Yale SH (2009). Reliability and feasibility of methods to quantitatively assess peripheral edema. Clin Med Res.

[9] Rabe E, Stucker M, Ottillinger B (2010). Water displacement leg volumetry in clinical studies--a discussion of error sources. BMC Med Res Methodol.

[10] Commean PK, Tuttle LJ, Hastings MK, Strube MJ, Mueller MJ (2011). Magnetic resonance imaging measurement reproducibility for calf muscle and adipose tissue volume. J Magnetic Reson Imaging.

[11] Sanders JE, Fatone S (2011). Residual limb volume change: systematic review of measurement and management. J Rehabil Res Dev.

[12] Cau N, Galli M, Cimolin V, Grossi A, Battarin I, Puleo G (2018). Quantitative comparison between the laser scanner three-dimensional method and the circumferential method for evaluation of arm volume in patients with lymphedema. J Vasc Surg Venous Lymphat Disord.

[13] Medical Compression Hoisery: RAL Deutsches Institut für Gütersicherung und Kennzeichnung e.V.; 2008 [Available from: http://www.gzg-kompressionsstruempfe.de/uploads/media/RAL_GZ_387_englische_Version.pdf.

[14] Hjermstad MJ, Fayers PM, Haugen DF, Caraceni A, Hanks GW, Loge JH (2011). Studies comparing numerical rating scales, verbal rating scales, and visual analogue scales for assessment of pain intensity in adults: a systematic literature review. J Pain Symptom Manag.

[15] Pichonnaz C, Bassin JP, Lecureux E, Currat D, Jolles BM (2015). Bioimpedance spectroscopy for swelling evaluation following total knee arthroplasty: a validation study. BMC Musculoskelet Disord.

[16] Gao FQ, Li ZJ, Zhang K, Huang D, Liu ZJ (2011). Risk factors for lower limb swelling after primary total knee arthroplasty. Chin Med J.

[17] Jakobsen TL, Christensen M, Christensen SS, Olsen M, Bandholm T (2010). Reliability of knee joint range of motion and circumference measurements after total knee arthroplasty: does tester experience matter?. Physiother Res Int.

[18] Stocker B, Babendererde C, Rohner-Spengler M, Muller UW, Meichtry A, Luomajoki H (2018). Effective therapy to reduce edema after total knee arthroplasty multi-layer compression therapy or standard therapy with cool pack - a randomized controlled pilot trial. Pflege..

[19] Holm B, Kristensen MT, Bencke J, Husted H, Kehlet H, Bandholm T (2010). Loss of knee-extension strength is related to knee swelling after total knee arthroplasty. Arch Phys Med Rehabil.

[20] Reich-Schupke S, Feldhaus F, Altmeyer P, Mumme A, Stucker M (2013). Efficacy and comfort of medical compression stockings with low and moderate pressure six weeks after vein surgery. Phlebology.

[21] Geerts WH, Pineo GF, Heit JA, Bergqvist D, Lassen MR, Colwell CW (2004). Prevention of venous thromboembolism: the seventh ACCP conference on antithrombotic and thrombolytic therapy. Chest..

[22] Agu O, Hamilton G, Baker D (1999). Graduated compression stockings in the prevention of venous thromboembolism. Br J Surg.

[23] Shalhoub J, Norrie J, Baker C, Bradbury AW, Dhillon K, Everington T (2017). Graduated compression stockings as an adjunct to low dose low molecular weight heparin in venous thromboembolism prevention in surgery: a multicentre randomised controlled trial. Eur J Vasc Endovasc Surg.

[24] Mauck KF, Froehling DA, Daniels PR, Dahm DL, Ashrani AA, Crusan DJ (2013). Incidence of venous thromboembolism after elective knee arthroscopic surgery: a historical cohort study. J Thromb Haemost.

[25] Maletis GB, Inacio MC, Reynolds S, Funahashi TT (2012). Incidence of symptomatic venous thromboembolism after elective knee arthroscopy. J Bone Joint Surg Am.

[26] Sun Y, Chen D, Xu Z, Shi D, Dai J, Qin J (2014). Incidence of symptomatic and asymptomatic venous thromboembolism after elective knee arthroscopic surgery: a retrospective study with routinely applied venography. Arthroscopy..

[27] Boehler K, Kittler H, Stolkovich S, Tzaneva S (2014). Therapeutic effect of compression stockings versus no compression on isolated superficial vein thrombosis of the legs: a randomized clinical trial. Eur J Vasc Endovasc Surg.

[28] Flevas DA, Megaloikonomos PD, Dimopoulos L, Mitsiokapa E, Koulouvaris P, Mavrogenis AF (2018). Thromboembolism prophylaxis in orthopaedics: an update. EFORT Open Rev.

[29] Wirth T, Schneider B, Misselwitz F, Lomb M, Tuylu H, Egbring R (2001). Prevention of venous thromboembolism after knee arthroscopy with low-molecular weight heparin (reviparin). Results of a randomized controlled trial Arthroscopy.

[30] Perrin M, Guex JJ (2000). Edema and leg volume: methods of assessment. Angiology..

